# Evaluation of clinical formalin‐fixed paraffin‐embedded tissue quality for targeted‐bisulfite sequencing

**DOI:** 10.1111/pin.13054

**Published:** 2020-12-17

**Authors:** Hideki Ohmomo, Shohei Komaki, Kanako Ono, Yoichi Sutoh, Tsuyoshi Hachiya, Eri Arai, Hiroyuki Fujimoto, Teruhiko Yoshida, Yae Kanai, Makoto Sasaki, Atsushi Shimizu

**Affiliations:** ^1^ Iwate Tohoku Medical Megabank Organization, Iwate Medical University, 1‐1‐1 Idaidori, Yahaba, Shiwa Iwate 028‐3694 Japan; ^2^ Department of Pathology Keio University School of Medicine, 35 Shinanomachi, Shinjuku Tokyo 160‐8582 Japan; ^3^ Division of Molecular Pathology National Cancer Center Research Institute, 5‐1‐1, Tsukiji, Chuo Tokyo 104‐0045 Japan; ^4^ Department of Urology National Cancer Center Hospital, 5‐1‐1 Tsukiji, Chuo Tokyo 104‐0045 Japan; ^5^ Department of Clinical Genomics National Cancer Center Research Institute, 5‐1‐1 Tsukiji, Chuo Tokyo 104‐0045 Japan; ^6^ Division of Ultrahigh Field MRI Institute for Biomedical Sciences, Iwate Medical University, 1‐1‐1 Idaidori, Yahaba, Shiwa Iwate 028‐3694 Japan

**Keywords:** DNA methylation, Epigenetics, Formalin‐fixed paraffin‐embedded tissue, Fresh frozen tissue, Targeted‐bisulfite sequencing

## Abstract

Formalin‐fixed paraffin‐embedded (FFPE) tissues are promising biological resources for genetic research. Recent improvements in DNA extraction from FFPE samples allowed the use of these tissues for multiple sequencing methods. However, fundamental research addressing the application of FFPE‐derived DNA for targeted‐bisulfite sequencing (TB‐seq) is lacking. Here, we evaluated the suitability of FFPE‐derived DNA for TB‐seq. We conducted TB‐seq using FFPE‐derived DNA and corresponding fresh frozen (FF) tissues of patients with kidney cancer and compared the quality of DNA, libraries, and TB‐seq statistics between the two preservation methods. The approximately 600‐bp average fragment size of the FFPE‐derived DNA was significantly shorter than that of the FF‐derived DNA. The sequencing libraries constructed using FFPE‐derived DNA and the mapping ratio were approximately 10 times and 10% lower, respectively, than those constructed using FF‐derived DNA. In the mapped data of FFPE‐derived DNA, duplicated reads accounted for > 60% of the obtained sequence reads, with lower mean on‐target coverage. Therefore, the standard TB‐seq protocol is inadequate for obtaining high‐quality data for epigenetic analysis from FFPE‐derived DNA, and technical improvements are necessary for enabling the use of archived FFPE resources.

**Abbreviations**
DNAm: DNA methylation
FFPE: Formalin‐fixed paraffin‐embedded tissues
FF: Fresh frozen tissue
gDNA: genomic DNA
TB‐seq: Targeted‐bisulfite sequencing

## INTRODUCTION

Formalin‐fixed paraffin‐embedded tissue (FFPE) has long been used as a permanent specimen preservation method in pathological and histological studies, including immunohistochemical^1^ and *in situ* hybridization^2^ studies. Formalin fixation results in cross‐linking of primary amines in nucleotides and amino acids, leading to DNA/RNA fragmentation and enzymatic activity inhibition.^3^ Thus, FFPE‐derived DNA is generally of low quality and considered unsuitable for omics analyses, including genome, transcriptome, and DNA methylation (DNAm) analyses. Recently, kits with improved efficiency for FFPE‐derived DNA extraction have become commercially available, allowing the preparation of sequencing libraries from FFPE samples. These technical improvements led to the use of FFPE‐derived DNA for whole‐genome sequencing,^4^ DNA capture sequencing,^5^, ^6^ RNA sequencing,^7^ and whole‐exome sequencing.^8^, ^9^

DNAm is an epigenetic mechanism that modulates gene expression. DNAm profiles possess developmental stage specificity and cell/tissue type specificity.^10^ Hence, DNAm patterns are considered novel biomarkers for clinical diagnosis before the onset and in the early stages of diseases. Array‐based DNAm analysis is widely used owing to the relatively low cost of DNAm profile analysis.^11^ High‐quality DNAm data can be obtained from 50 ng of FFPE‐derived genomic DNA (gDNA).^12^ However, because only a few CpG sites can be analyzed using the DNAm profile analysis, most potential biomarkers remain unsurveyed.

Recently, we identified a novel DNAm biomarker for severe aortic valve stenosis using TB‐seq and demonstrated that TB‐seq is useful in searching for novel DNAm biomarkers.^13^ FFPE‐derived DNA represents a vast pathological resource that could be utilized for DNAm analysis. However, there is no established approach for the use of FFPE‐derived DNA for DNAm analysis. Here, we focused on TB‐seq and compared the quality of gDNA, libraries, and TB‐seq statistics obtained from fresh frozen (FF) or FFPE samples.

## MATERIALS AND METHODS

### Samples and ethics

The FFPE and FF samples were prepared from the same cancerous and non‐cancerous tissue samples of the same individual. FFPE and FF samples were microscopically and macroscopically observed by a pathologist and collected from non‐necrotic areas. FFPE samples were fixed using 10% neutral‐buffered formalin for 1–3 days in accordance with the Japanese Society of Pathology Guidelines for the Handling of Pathological Tissue Samples for Genomic Research.^14^ Written informed consent was obtained before treatment. This study was approved by the Ethics Committee of the National Cancer Center, Tokyo, Japan, and Keio University.

### DNA extraction, library preparation, and sequencing

**Figure 1:**
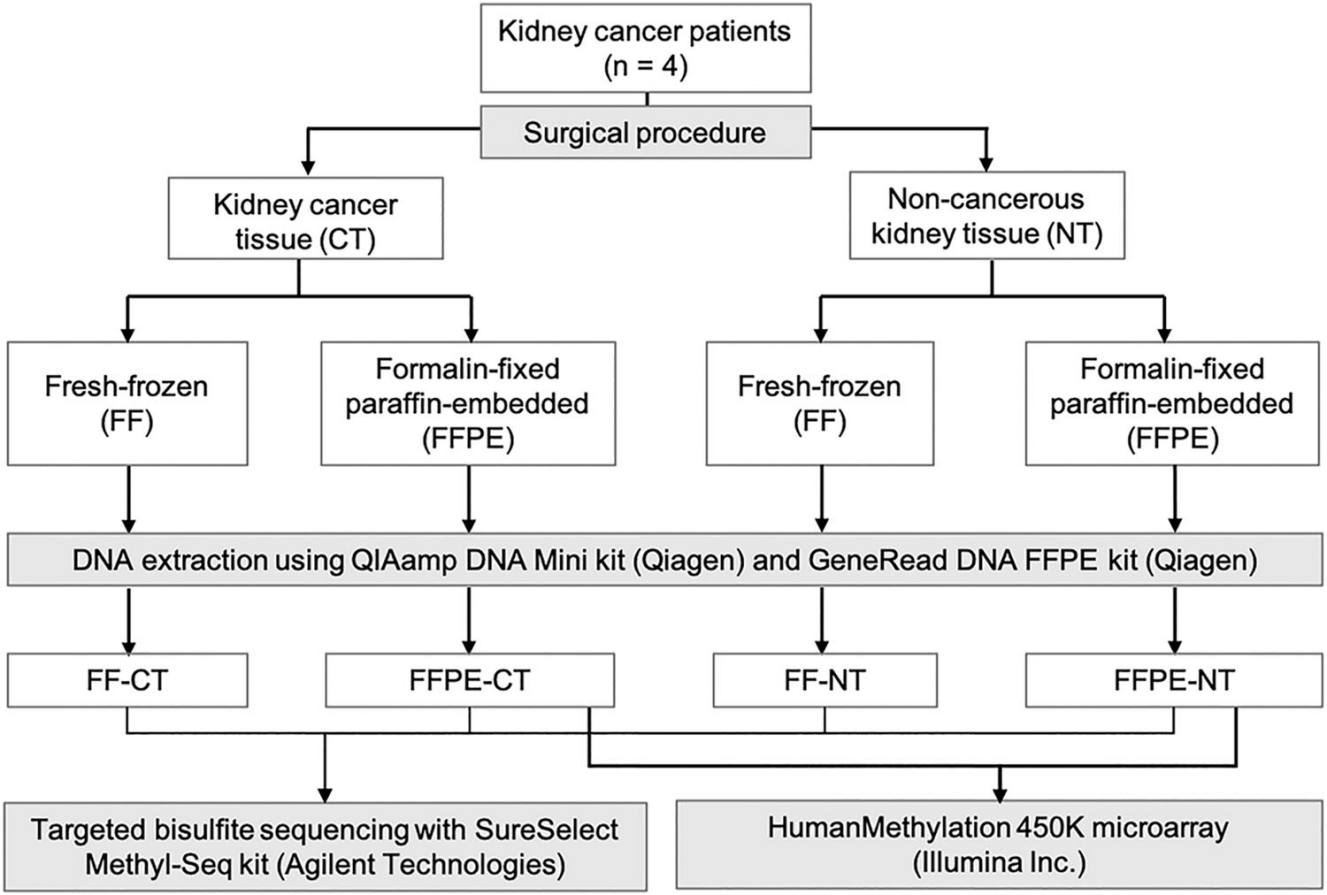
Summary of the workflow and study design.

The workflow summary is shown in Figure 1. FFPE‐derived DNA was prepared as previously reported.^12^ gDNA was extracted from FF tissue using the QIAamp DNA Mini kit (Qiagen, Hilden, Germany). A GeneRead DNA FFPE kit (Qiagen) was used to extract gDNA from FFPE tissue. DNA yield and quality were assessed using the Agilent 2200 TapeStation (Agilent Technologies, Santa Clara, CA, USA). Absorbance was measured using a Nanodrop 2000/2000c Spectrophotometer (Thermo Fisher Scientific, Waltham, MA, USA).

To prepare DNA for library construction, 1 μg of gDNA was sheared to a size range of 150–175 bp using the focused‐ultrasonicator (Covaris Inc., Woburn, MA, USA). Sequencing libraries were prepared with Agilent SureSelect^XT^ Human Methyl‐Seq Capture Library and Reagent kit (Agilent) according to manufacturer's instructions. Finally, all libraries were treated with sodium bisulfite using the EZ DNA Methylation‐Gold Kit (Zymo Research, Irvine, CA, USA).

Library fragment size was measured using a D1000 ScreenTape and Reagents Kit (Agilent), and the yields were quantified by real‐time PCR using Kapa Library Quantification Kit (NIPPON Genetics Co., Ltd., Tokyo, Japan). The libraries were sequenced (2 × 125 bp) using a HiSeq2500 (Illumina Inc., San Diego, CA, USA).

### DNAm profiling using TB‐seq

DNAm profiles were evaluated according to bioinformatics workflows previously described.^15^, ^16^ First, the quality of raw data was assessed using FastQC (v0.11.5). Short reads (<20 bp) were removed, and adapters were trimmed using Trim Galore (v0.4.2). The remaining reads were aligned to a human reference genome (hs37d5) using Novoalign (v3.6.5). Subsequently, the files were processed by clipping overlaps and removing duplicate reads using several bioinformatics tools such as SAMtools (v1.3.1), Picard‐tools (v2.7.1), and BamTools (v2.4.0). Finally, sequence statistics were calculated using R (v3.3.1).

### HumanMethylation 450 K array analysis

Fifty nanograms of FFPE‐derived gDNA was treated with bisulfite conversion reagent and examined using the HumanMethylation 450 K array (Illumina). The number of detected CpGs was calculated using the GenomeStudio software.

## RESULTS

To ascertain whether FFPE‐derived DNA is suitable for TB‐seq analysis, we compared the quality of gDNA, libraries, and sequence statistics of FF and FFPE samples collected from four kidney cancer patients.

### Comparison of sample quality between FFPE and FF tissues

**Figure 2:**
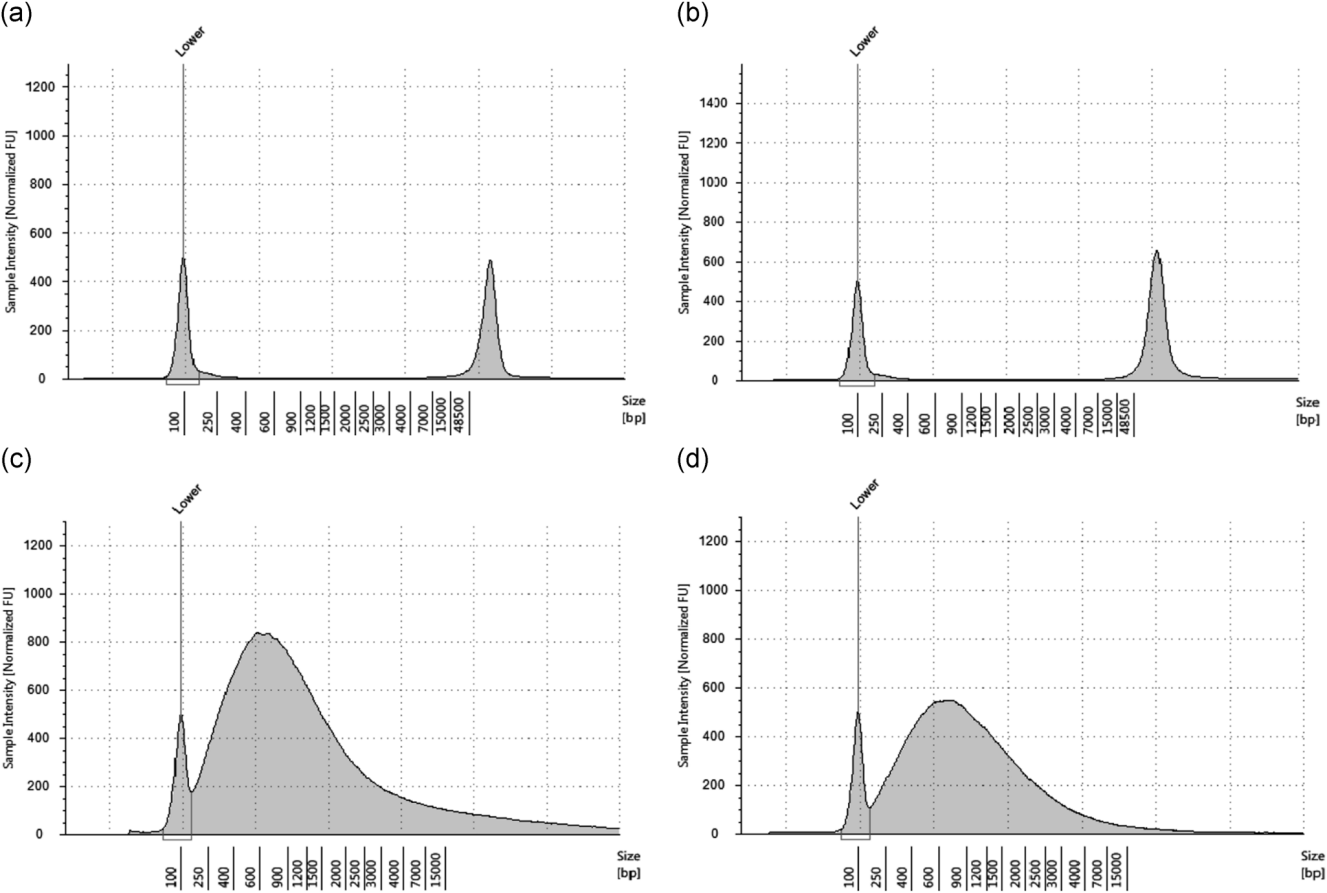
Comparison of genomic DNA quality using TapeStation 2200. Electropherograms of gDNA from FF‐CT (a), FF‐NT (b), FFPE‐CT (c), and FFPE‐NT (d). The x‐axis and the y‐axis represent the fragment size (bp) of gDNA and sample intensity (normalized FU), respectively. FF‐CT, fresh‐frozen cancer tissue; FF‐NT, fresh‐frozen non‐cancer tissue; FFPE‐CT, formalin‐fixed paraffin‐embedded cancer tissue; FFPE‐NT, formalin‐fixed paraffin‐embedded non‐cancer tissue.

The absorbance ratio at 260 and 280 nm (A_260/280_) of the isolated DNA solutions from FF and FFPE samples ranged between 1.86 and 1.92 (Table 1), indicating high quality. The A_260/230_ ratio of FF‐derived DNA ranged between 1.73 and 2.10, indicating relative purity. However, the A_260/230_ ratio of FFPE‐derived DNA ranged between 1.05 and 1.23 (Table 1). The mean FF‐derived gDNA peak size was > 50,000 bp (Figure 2A and B), whereas it was approximately 550 bp for the FFPE‐derived gDNA (Figure 2C and D). The DNA integrity number of the FFPE‐derived DNA was 1.6, indicating extremely low quality. Sequencing libraries generated from FFPE‐derived DNA yielded approximately 4.4–5.0 nM, 10 times fewer than that of FF‐derived DNA (Table 1).

**Table 1:** Comparison of sample quality from genomic DNA with sequencing libraries

Experiment step	Instruments	Category	FF‐CT	FF‐NT	FFPE‐CT	FFPE‐NT
Genomic DNA	Nanodrop	Conc. (ng/µL)	28.9 ± 9.6	24.6 ± 12.6	379.6 ± 61.1#	270.3 ± 17.5#
		A260/280	1.92 ± 0.07	1.86 ± 0.04	1.87 ± 0.01	1.88 ± 0.01
		A260/230	1.73 ± 0.14	2.10 ± 0.18	1.23 ± 0.09	1.05 ± 0.03
	TapeStation	Conc. (ng/µL)	9.1 ± 2.0	11.8 ± 5.5	65.7 ± 29.2	21.1 ± 15.9
		Peak size	52,586 ± 12,841	60,000<	535 ± 90	554 ± 94
		DIN	9.8 ± 0.2	9.6 ± 0.3	1.6 ± 0.1	1.6 ± 0.3
Genomic DNA after fragmentation	TapeStation	Conc. (ng/µL)	26.9 ± 4.8	15.8 ± 5.9	11.2 ± 0.8	11.7 ± 1.1
		Average size (bp)	160.3 ± 3.6	165.3 ± 5.9	213.0 ± 7.8	223.0 ± 9.0
Sequencing libraries	TapeStation	Conc. (ng/µL)	9.0 ± 2.0	4.5 ± 2.1	0.6 ± 0.1	0.7 ± 0.2
		Average size (bp)	304.0 ± 3.2	305.5 ± 5.6	280.8 ± 4.6	281.8 ± 5.3
		Molarity (nM)	47.3 ± 10.1	24.0 ± 11.0	4.0 ± 0.3	4.3 ± 1.1
	qPCR	Molarity (nM)	71.7 ± 24.2	38.9 ± 21.1	4.4 ± 1.0	5.0 ± 2.2

^#^ Because DNA from FFPE‐CT and FFPE‐NT was concentrated after the DNA extraction, the concentration of gDNA from FFPE was 10 times higher than in FF. **FF‐CT**, fresh‐frozen cancer tissue; **FF‐NT**, fresh‐frozen non‐cancerous tissue; **FFPE‐CT**, formalin‐fixed paraffin‐embedded cancer tissue; **FFPE‐NT**, formalin‐fixed paraffin‐embedded non‐cancerous tissue; **Conc.**, concentration; **A**_**260/280**_, Absorbance ratio between 260 nm and 280 nm; **A**_**260/230**_, Absorbance ratio between 260 nm and 230 nm; **DIN**, DNA integrity number.

### Comparison of library quality and sequencing statistics between FFPE and FF tissues

The amounts of raw data obtained from FFPE libraries were approximately 10% lower than those from FF libraries (paired *t*‐test *P* = 0.0042) (Table 2), although the concentration of libraries used for sequencing was the same (Table 2). The ratios of mapped reads to the reference genome were approximately 95% in FF libraries and 85% in FFPE libraries, similar to that of high‐quality, optimized, reduced representation bisulfite sequencing using 50 ng of FFPE‐derived DNA.^17^ Nevertheless, the ratio of PCR duplicate reads was higher in FFPE libraries than in FF libraries (> 60% vs. 10–20%) (Table 2). The average ratio of on‐target reads for FF samples was approximately 80%, and the mean on‐target coverage was over 30×. However, for FFPE samples, we observed a <70% on‐target reads ratios and a mean on‐target coverage of 7.2–7.3× (Table 2). Of the 3.7 million CpGs designed for Agilent ready‐made probes, the number of detected CpGs was more than 3.6 million in FF (>97%) and only 3 million in FFPE (approximately 81%) (Table 2).

**Table 2:** Sequence statistics

	FF‐CT	FF‐NT	FFPE‐CT	FFPE‐NT
Median insert size	145.3 ± 1.1	147.8 ± 3.3	127.3 ± 2.9	128.5 ± 2.7
Total raw reads	60,701,176 ± 2,611,637	61,386,410 ± 3,225,703	56,042,463 ± 3,217,046	52,955,491 ± 2,097,483
Mapped reads	57,663,943 ± 2,875,484	58,101,677 ± 2,583,479	47,686,960 ± 3,607,514	45,190,024 ± 1,783,127
% of mapped reads	95.0 ± 0.9	94.7 ± 0.8	85.0 ± 2.4	85.3 ± 1.1
Remaining reads after clipping	52,155,448 ± 3,444,210	45,920,354 ± 5,464,927	13,590,626 ± 1,911,843	13,689,569 ± 3,818,050
% of duplicated reads	9.6 ± 2.1	21.0 ± 8.4	64.5 ± 4.4	61.5 ± 2.6
Genome size	3,137,503,007	3,137,503,007	3,137,503,007	3,137,503,007
Mapped bases passed through a filter	3,974,470,410 ± 287,091,192	3,553,923,182 ± 414,817,717	894,631,300 ± 146,916,493	918,060,184 ± 277,587,019
On‐target bases (%)	3,178,559,259 ± 214,364,542 (80.0 ± 0.5)	2,824,621,506 ± 337,411,613 (79.5 ± 0.7)	608,073,556 ± 100,282,511 (68.0 ± 0.9)	619,774,900 ± 202,754,019 (67.1 ± 1.6)
Near‐target bases# (%)	547,441,692 ± 47,772,892 (13.8 ± 0.5)	495,694,119 ± 62,502,784 (13.9 ± 0.5)	87,521,342 ± 16,770,315 (9.8 ± 0.4)	91,630,733 ± 30,598,132 (9.9 ± 0.3)
Off‐target bases (%)	248,469,459 ± 27,983,602 (6.2 ± 0.3)	233,607,557 ± 20,337,832 (6.6 ± 0.3)	199,036,402 ± 31,488,025 (22.3 ± 0.9)	248,469,459 ± 27,983,605 (23.0 ± 1.9)
Mean on‐target coverage	37.6 ± 2.6	33.5 ± 4.0	7.2 ± 1.2	7.3 ± 2.4
Number of CpG sites on Methyl‐Seq probe	~ 3.7 Million	~ 3.7 Million	~ 3.7 Million	~ 3.7 Million
Number of CpG sites (1× depth)	3,682,842 ± 38,116	3,622,934 ± 51,977	3,040,326 ± 130,104	2,937,087 ± 127,111
Number of CpG sites (>6× depth)	2,884,711 ± 18,924	2,876,447 ± 26,253	1,611,751 ± 254,405	1,517,406 ± 356,333

^#^ Near‐target bases are defined as the detected bases mapped within 1 kb upstream or downstream of Agilent ready‐made probes. **FF‐CT**, fresh‐frozen cancer tissue; **FF‐NT**, fresh‐frozen non‐cancerous tissue; **FFPE‐CT**, formalin‐fixed paraffin‐embedded cancer tissue; **FFPE‐NT**, formalin‐fixed paraffin‐embedded non‐cancerous tissue.

### Number of detected CpGs of FFPE samples in HM450 microarray analysis

To compare the results of the quality assessment of the TB‐Seq in this study with those of the microarray analysis, HM450 microarray analysis was performed on FFPE samples. The results showed that more than 97% (*P* < 0.05) or more than 95% (*P* < 0.01) of the CpGs were detectable in the FFPE samples (Table 3).

**Table 3:** Number of detected CpGs from FFPE samples using the HumanMethylation450 microarray analysis

	FFPE‐CT	FFPE‐NT
Number of CpGs on HM450 microarray	485,577	485,577
Number of detected CpGs (*P* < 0.05)	476,893 ± 2,622	474,098 ± 5,463
% of detected CpGs (*P* < 0.05)	98.2 ± 0.5	97.6 ± 1.1
Number of detected CpGs (*P* < 0.01)	464,680 ± 3,181	464,286 ± 6,550
% of detected CpGs (*P* < 0.01)	95.7 ± 0.7	95.6 ± 1.3

**FFPE‐CT**, formalin‐fixed paraffin‐embedded cancer tissue; **FFPE‐NT**, formalin‐fixed paraffin‐embedded non‐cancerous tissue; **HM450**, HumanMethylation450.

## DISCUSSION

FFPE‐derived DNA is a rich source of genetic material for molecular diagnostic and pathological studies. Although FFPE‐derived DNA has recently been available for analysis in multiple sequencing methods,^4^, ^5^, ^6^, ^7^, ^8^, ^9^ sequencing‐based DNA methylation analysis is still largely unreported. Contrarily, DNA methylation analysis using microarrays is widely used worldwide, and microarray analysis of small amounts of FFPE‐derived DNA as small as 50 ng has been established, as reported by Ohara *et al*.^12^ Our microarray analysis results are consistent with a previous report^12^ and could obtain high‐quality data from FFPE‐derived DNA.

Our TB‐seq results indicate that the quality of FFPE‐derived DNA is lower than that of FF‐derived DNA, indicating organic contaminants in the isolated DNA solution and fragmentation of gDNA. These results are consistent with those of previous reports.^18^, ^19^ Furthermore, the organic contamination and fragmentation of gDNA may have caused PCR bias and increased the fraction of duplicate reads in the FFPE sample. The high percentage of duplicate reads seen in PCR amplification of FFPE libraries indicated that the amount of input gDNA used in the existing TB‐seq protocol was not enough to ensure the complexity of the libraries. In addition, to obtain the same level of on‐target coverage from the FFPE and FF samples, approximately five times the data is necessary, which is not cost‐effective.

In conclusion, the existing protocol for TB‐seq is insufficient for preparing sequencing libraries from FFPE‐derived DNA for general DNAm analysis. Technical improvements limiting the DNA fragmentation are necessary before reliably using the archived FFPE resources for this analysis. This approach will help facilitate epigenetic research using FFPE tissue archives and identify novel DNAm biomarkers for various diseases and environmental exposures.

## DISCLOSURE STATEMENT

There are no potential conflicts of interest to disclose.

## AUTHOR CONTRIBUTIONS

Conception and design: HO, YK, and AS.

Acquisition of data: HO and KO.

Analysis and interpretation of data: HO, SK, and KO.

Writing, review, and/or revision of the manuscript: HO, SK, KO, YS, TH, EA, HF, TY, YK, MS, and AS.

Administrative, technical, or material support: HO, KO, EA, HF, TY, and YK
